# Operational standard for the clinical use of Lecanemab in Alzheimer’s Disease

**DOI:** 10.1002/alz70859_097091

**Published:** 2025-12-25

**Authors:** Jin Wang, Shan Wei, Qiumin Qu

**Affiliations:** ^1^ The First Affiliated Hospital of Xi'an Jiaotong University, Xi'an, Shaanxi China

## Abstract

**Background:**

Lecanemab is approved by FDA for early Alzheimer’s disease (AD) treatment, and has entered China by the end of June 2024. However, adverse events may occur with Lecanemab including amyloid related imaging abnormalities (ARIA) and infusion reactions. To help clinicians administer Lecanemab, we establish an operational standard for the clinical use of Lecanemab in AD patients.

**Method:**

Referring to the instructions of Lecanemab and the Clarity Phase III clinical trial protocol, combined with the recommendations of Chinese clinical experts, establish an operational standard.

**Result:**

The target population for Lecanemab is: 1. Mild cognitive impairment or mild dementia caused by Alzheimer's disease; 2. PET shows deposition of Aβ in the brain, or a decrease Aβ_1‐42_ or Aβ _1‐42_/Aβ _1‐40_ ratio in cerebrospinal fluid; 3. Moderate or severe AD, strongly requested to use Lecanemab by family members. People with caution to use Lecanemab: 1. History of cerebral hemorrhage, especially cerebral hemorrhage caused by amyloid angiopathy; 2. Susceptibility‐weighted imaging(SWI) shows more than 5 microbleeds in the cerebral cortex; 3. Currently using warfarin, low molecular weight heparin, or new oral anticoagulant drugs; 4. Individuals with an allergic constitution and a history of severe allergies. Preparation before the first treatment: 1. Complete imaging evaluation: Brain MRI scan+T2‐FAIR+SWI within 6 months; 2. Cognitive function assessment: MMSE, CDR‐SB, ADAS‐cog, ADCS‐ADL;3. Detecting ApoE genotype; 4. Sign the informed consent form. Treatment process: 1. Patient completes hospitalization procedures; 2. Routine examinations: blood routine, liver and kidney function, electrolytes, myocardial enzyme spectrum, electrocardiogram; 3. Collect venous blood for the determination of plasma Aβ_1‐42_, Aβ_1‐40_, and P‐tau181; 4. Monitor ECG, blood pressure, and finger pulse oxygen; 5. Calculate the dose of Lecanemab based on 10mg/kg, add it to 250ml of 0.9% saline, and intravenously drip it with an infusion pump within 1 hour; During the infusion period, closely monitor the condition and measure every 15 minutes until 3 hours after the infusion is completed; 7. Record adverse reactions.

**Conclusion:**

We establish an operational standard for clinical use of Lecanemab in the AD treatment. It is helpful in clinical practice for patient selection, management, observation of efficacy and adverse reactions.

